# Health dialogue intervention versus opportunistic screening in primary care for type 2 diabetes and cardiovascular disease prevention in settings with low socioeconomic status (DETECT): study protocol for a pragmatic cluster-randomized trial

**DOI:** 10.1186/s13063-024-08533-8

**Published:** 2024-10-12

**Authors:** Marcel Ballin, Moa Backman Enelius, Samira Dini, Maria Rosaria Galanti, Maria Hagströmer, Emelie Heintz, Anton Lager, Antonio Ponce de Leon, Lena Lundh, Camilla Nystrand, Christina Walldin, Hanna Augustsson

**Affiliations:** 1 Centre for Epidemiology and Community Medicine, Region Stockholm, Stockholm, Sweden; 2 Department of Public Health and Caring Sciences, Clinical Geriatrics, Uppsala University, Uppsala, Sweden; 3 Department of Global Public Health, Karolinska Institutet, Stockholm, Sweden; 4 Division of Physiotherapy, Department of Neurobiology, Care Sciences and Society, Karolinska Institutet, Stockholm, Sweden; 5 Academic Primary Health Care Centre, Region Stockholm, Stockholm, Sweden; 6 Department of Health Promoting Science, Sophiahemmet University, Stockholm, Sweden; 7 Stockholm Center for Health Economics, Region Stockholm, Stockholm, Sweden; 8 Department of Learning, Informatics, Management and Ethics, Karolinska Institutet, Stockholm, Sweden; 9 Division of Family Medicine and Primary Care, Department of Neurobiology, Care Sciences and Society, Karolinska Institutet, Stockholm, Sweden; 10 Procome Research Group, Department of Learning, Informatics, Management and Ethics, Medical Management Centre, Karolinska Institutet, Stockholm, Sweden

**Keywords:** Screening, Health check, Health promotion, Lifestyle, Prevention, Primary care

## Abstract

**Background:**

Meta-analyses of randomized trials suggest that health checks and health promotion interventions targeting behavior change in primary care do not prevent cardiovascular morbidity and mortality in the general population. However, whether such interventions are more effective in high-risk populations, such as people living in low socioeconomic settings, remains unclear, as they have been poorly represented in previous trials. Therefore, we aim to evaluate the effectiveness, cost-effectiveness, and implementation of systematic screening followed by an individually oriented, lifestyle-focused, health dialogue intervention for prevention of type 2 diabetes and cardiovascular disease, as compared to opportunistic screening, in primary care in socioeconomically disadvantaged areas.

**Methods:**

Using an overall pragmatic approach and a cluster-randomized design with two arms, we aim to enroll 3000 participants aged 50–59 years from 30 primary care centers (PCCs) with an above-average level of Care Need Index in Stockholm Region, Sweden. PCCs will be randomized (1:1) either to a health dialogue intervention, which includes inviting enlisted patients to a systematic screening of risk factors followed by an individually oriented lifestyle-focused health dialogue, or to opportunistic screening, which includes screening patients for a smaller set of risk factors during an appointment at their PCC taking place for other reasons. The main outcome will be change in systolic blood pressure during 6- and 12-month follow-ups. Additional short-term outcomes will be changes in other biological risk factors, health-related quality-of-life, and lifestyle habits, as well as process and implementation outcomes, and unintended side effects. The long-term effect on type 2 diabetes and cardiovascular disease incidence and mortality will be examined using regional and nationwide registers. Changes in systolic blood pressure and other health outcomes will be analyzed using mixed-effect generalized linear modeling and mixed-effect Cox regression to capture variability between and within PCCs. A health economic evaluation will assess resource use and costs in the short- and long-term.

**Discussion:**

This trial of lifestyle-focused health dialogues and opportunistic screening in primary care in socioeconomically disadvantaged areas in the largest region of Sweden has the potential to yield valuable insights that could support evidence-based policymaking.

**Trial registration:**

ClinicalTrials.gov (NCT06067178). Prospectively registered September 27, 2023.

**Supplementary Information:**

The online version contains supplementary material available at 10.1186/s13063-024-08533-8.

## Background

Type 2 diabetes (T2D) and cardiovascular disease (CVD) are estimated to be prevalent in more than 500 and 620 million people respectively, leading to substantial disability, mortality, and economic burden [1–3]. A substantial proportion of the disease burden can be attributed to modifiable risk factors such as hypertension, hyperglycemia, dyslipidemia, obesity, unhealthy diet, smoking, and physical inactivity [1, 4]. Among the indirect favorable consequences of preventive interventions targeting such factors could be a decreased demand imposed on healthcare and welfare systems. Because modifiable risk factors cluster among people with low socioeconomic status [5], targeted interventions may be particularly important to reduce heath inequality.

Primary care has been proposed as a promising arena for the delivery of preventive interventions given its large reach [6]. Some countries, such as Sweden and the UK, have incorporated different types of population-based screening programs and health assessments into primary care [7, 8]. Unfortunately, the scientific evidence supporting large-scale implementation of such programs is weak, as reported by numerous Cochrane meta-analyses of randomized trials. One meta-analysis found limited evidence that systematic screening (predetermined and systematic process for selecting and inviting participants to a screening program) compared to opportunistic screening (sporadically occurring risk assessment without systematic invitation) can have favorable effects on some risk factors such as blood pressure but not on cardiovascular events [9]. Similarly, another meta-analysis concluded that systematically offered general health checks have no effects on cardiovascular events as compared to no health checks [10]. Both of these meta-analyses focused on trials in healthy adults from the general population, making generalizability to high-risk populations uncertain.

Another possibility is that screening alone might not be sufficient to induce improvements in risk factors, thereby limiting its ability to subsequently reduce clinical outcomes, which would suggest the need for more intense interventions. For example, one meta-analysis of health promotion interventions aimed at behavior change found that although such interventions do not reduce cardiovascular events in general populations, they might be effective in certain risk groups, i.e., people with hypertension and T2D [11]. Similarly, another meta-analysis of health promotion interventions targeting risk groups, in this case people living in low- and middle-income settings, suggested beneficial effects on some surrogate measures such as blood pressure. Yet, heterogeneity was substantial and there was not enough evidence to determine the effect on cardiovascular events and lifestyle habits [12], lending the authors to request further trials.

In Sweden, a prevention program has been developed in primary care since the mid-1980s [13, 14]. The program includes systematic screening of T2D and CVD risk factors followed by a person-centered lifestyle health dialogue based on the individual risk profile. The screening assessment includes questionnaires assessing a wide range of factors including medical history, medications, general health, mental health, heredity, living situation, quality-of-life, and lifestyle habits as well as blood tests of biological risk factors, blood pressure measurement, and anthropometric measurements. A recent systematic review suggested that the Swedish prevention program reduces all-cause mortality, cardiovascular mortality, blood pressure, cholesterol, blood glucose, waist circumference, and body mass index, while positively influencing dietary habits [15]. No effects of the program were detected for physical activity, tobacco use, and alcohol consumption. It should, however, be noted that the quality of the underlying evidence ranged from low-to-moderate, and six out of seven studies included in the review were observational. Moreover, the Swedish program has neither been implemented or evaluated in settings specifically targeting socioeconomically disadvantaged settings nor has it been compared to other preventive interventions. This would be important given the typically lower participation rates observed in these areas [16].

Based on the available evidence outlined above, it could be hypothesized that an intervention acting on the results of a screening assessment, i.e., person-centered lifestyle health dialogues, might be beneficial if it targets specific populations with a high burden of risk factors such as people in low-socioeconomic settings, rather than the general population. Targeting risk groups instead of a whole population may not only be more effective and cost-effective [17] but can also be important for tackling health inequalities. Considering the scarcity of evidence regarding the effectiveness, cost-effectiveness, and implementation of such preventive efforts delivered in primary care in areas with low socioeconomic status, a trial addressing this knowledge gap has the potential to contribute to the evidence base of these forms of interventions.

### Objectives

The overall aim of the *Health Dialogue intErvention versus opporTunistic scrEening in primary Care for Type 2 diabetes and cardiovascular disease prevention in settings with low socioeconomic status* (DETECT) trial is to evaluate the effectiveness, cost-effectiveness, and implementation of a systematic screening followed by an individually oriented, lifestyle-focused, health dialogue intervention (HDI) for prevention of T2D and CVD, as compared to opportunistic screening (OS), delivered in primary care in socioeconomically disadvantaged areas in Stockholm, Sweden. The primary outcome is change in systolic blood pressure, which is used as a surrogate outcome for the target outcome of CVD incidence. In 2022, there was a formal political decision that the Swedish prevention program for lifestyle-focused health dialogues should be implemented in primary care across the Stockholm Region as a pilot program. Consequently, the Health and Medical Care Administration commissioned Centre for Epidemiology and Community Medicine, and Academic Primary Health Care Centre, to implement and conduct an evaluation of the health dialogue pilot program. The original plan for the pilot program included a third arm receiving usual care. Unfortunately, due to limited initial responsiveness from primary care centers (PCCs), the trial could not be powered for a three-arm comparison. As a result, priority was given to comparing the two interventions described below.

The specific aims of this trial are to evaluate the following: (I) the long-term effects on T2D and CVD incidence and mortality among participants receiving HDI as compared to participants receiving OS; (II) the short-term effects on biological risk factors (blood pressure, cholesterol levels, glucose levels, body mass index) and health-related quality-of-life (HRQoL), among participants receiving HDI as compared to participants receiving OS; (III) the short-term effects on lifestyle habits (tobacco/nicotine usage, dietary habits, alcohol consumption, physical activity), among participants receiving HDI as compared to participants receiving OS; (IV) the potential effect modification by sex, socioeconomic status, and birth country, for all of the above; (V) the differences in resource use and associated costs between HDI and OS in the short and long terms; (VI) the costs per quality-adjusted life year (QALY) and per blood pressure target attained associated with HDI as compared to OS in the short term; and (VII) the cost per life year gained associated with HDI as compared to OS in the long-term.

Additionally, we will assess various implementation and process outcomes, including (I) the number of invited PCCs that agree to participate; (II) the adherence to the original intervention protocols by healthcare professionals; (III) the perceived acceptability, appropriateness, and feasibility of implementing the interventions among healthcare professionals; (IV) the barriers and facilitators for implementation of the interventions among healthcare professionals; (V) the proportion of patients choosing to participate among those invited and the reasons for declining participation among non-participants; (VI) the representativeness of participants as compared to the total eligible population based on socioeconomic characteristics; (VII) the case-finding rates, defined as the proportion of participants among whom elevated levels of biological risk factors are detected for the first time; and (VIII) the estimated time needed to treat (TNT).

## Methods

### Study design and setting

This is a pragmatic, population-based, superiority, cluster-randomized trial with two intervention arms, of which one serves as comparator. The first arm (HDI) consists of a systematic screening followed by an individually oriented lifestyle-focused health dialogue based on the risk profile obtained from the screening. The second arm (OS) consists of opportunistic screening and represents the comparator. The PCCs represent the clusters and unit of randomization, while patients and healthcare professionals at the PCCs represent units of observation.

This trial will be conducted within primary health care in the Stockholm Region in Sweden. The Stockholm Region has the largest population of the 21 regions in Sweden. The region had a total of 2,440,027 (23.2%) inhabitants in 2022, of which 313,924 were aged 50–59 years old, according to official, publicly available data from Statistics Sweden. In Sweden, primary care is part of the tax-funded health system, which is governed and managed by the 21 regions, which includes both public and private PCCs. In 2022, there were a total of 231 PCCs in Stockholm Region, of which 67 were public and 164 were private. The public and private PCCs are funded and governed according to the same rules but differ in how they are organized and managed. Both public and private PCCs will be included in this trial. The target population is individuals living in areas of low socioeconomic status, and we will therefore select PCCs based on their Care Need Index (CNI). The CNI is a social deprivation index which describes the expected risk of ill health within a patient population, based on socioeconomic and demographic factors [18].

### Trial reporting and ethical approval

Reporting of this protocol is guided by the Standard Protocol Items: Recommendations for Interventional Trials (SPIRIT)-Surrogate extension guidelines [19]. The results will be reported according to the Consolidated Standards of Reporting Trials (CONSORT) guidelines, with extensions to surrogate endpoints and cluster trials [20, 21]. Ethical approval of the trial has been granted by the Swedish Ethical Review Authority (Dnr. 2023–03001-01). The trial has been pre-registered at https://clinicaltrials.gov with reference number NCT06067178, where all items from the World Health Organization Trial Registration Data Set are available. Any major modifications of the protocol will be communicated to the trial registry.

### Sample size

Sample size calculations were based on change in systolic blood pressure as the main outcome measure. We considered a 5-mmHg reduction in systolic blood to be clinically relevant as meta-analyses of randomized trials show that such a reduction can reduce the risk of CVD by about 10% regardless of CVD history, including among people with normal to high-normal blood pressure [22], as well as specifically in middle-aged adults [23].

Based on systolic blood pressure data in Stockholm Region that were obtained from electronic medical records, the estimated variance of systolic blood pressure levels between PCCs (clusters) was equal to 2, and the variance between subjects within PCCs (individuals) was equal to 256. These settings give rise to the underlying variance structure of the data, essential for sample sizes calculations, namely a difference of 5 mmHg in systolic blood pressure between arms (VA = 12.5), variance between clusters, (VC = 2), and variance between individuals within PCCs (VE = 256). We calculated the number of clusters, the number of individuals, and the power of the sample using various combinations of VA, VC, and VE values close to those outlined above. For each calculation, we adjusted one parameter while holding the other two constant. We used the package clusterPower version 0.7.0 [24], R Statistical Software version 4.1.3 [25], for the sample size and/or power calculations for the different variance settings.

Accordingly, we calculated that based on 15 clusters in each arm, a minimum of 840 participants (*n* = 420 in each arm and *n* = 28 per cluster) would yield 80% power to detect a reduction of 5 mmHg systolic blood pressure in the HDI group as compared to OS. Rising power to 90% while keeping the number of clusters to 15 per arm would require a minimum of 1200 participants (*n* = 600 in each arm and *n* = 40 per cluster). Additionally, anticipating difficulties with cluster recruitment and retention, we also calculated that if the size of the cluster is increased to *n* = 69, a power of 80% would be obtained with 9 clusters per arm, and 90% power would be obtained with 11 clusters per arm. Because we expected difficulties with recruitment of PCCs and participant recruitment within PCCs, as well as subsequent attrition of the two, the goal was to recruit at least 30 PCCs (*n* = 15 in each arm) in Stockholm Region, each of which will aim to recruit *n* = 100 participants, yielding a maximum total study population of *N* = 3000.

### Study population, cluster eligibility, and cluster recruitment

**Fig. 1:**
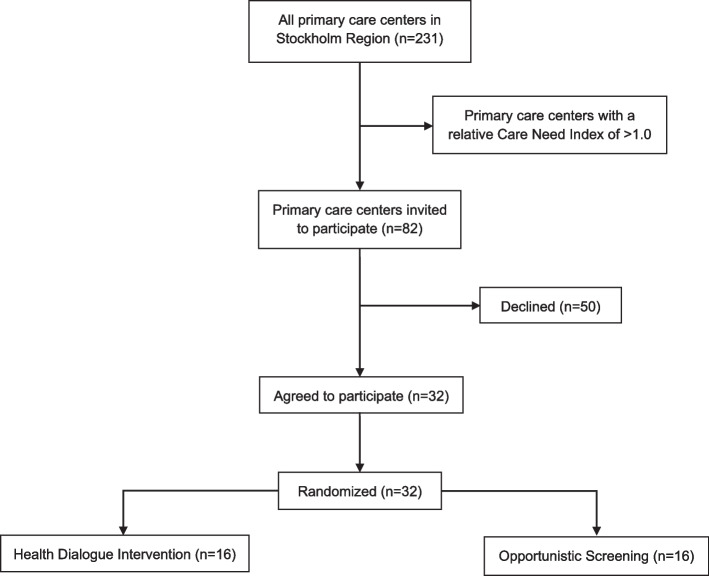
Flowchart of cluster eligibility, recruitment, and randomization

Eligible PCCs were those with the highest relative CNI in Region Stockholm. A lower CNI limit was set at 1.0 which represents the median value. First, the managers from the 50 PCCs with the highest relative CNI in the region were invited to a meeting where they were informed about the trial and had the opportunity to ask questions. After that, they had 14 days to decide whether they would like to sign up their PCC for participation. Because we did not reach the recruitment goal of 30 PCCs within these 14 days, the recruitment procedure was repeated with an additional 32 PCCs. Thus, a total of 82 PCCs were invited, and by 19 May 2023, the recruitment of PCCs was completed, resulting in a total of 31 PCCs that agreed to participate. One additional PCC informed us with their agreement to participate after the 14-day deadline was completed (31 October 2023) and after the randomization (see description below) of the 31 PCCs. However, the steering committee decided to include this PCC and randomize it individually. The relative CNI for the 32 randomized PCCs range from 2.83 to 1.07. A flowchart of cluster eligibility, recruitment, and randomization is presented in Fig. 1.

### Randomization and blinding

A matched-pair cluster randomization was performed using the R Statistical Software 4.1.3 [25], employing the *rbinom* function to generate computer-generated random numbers. The underlying code is available in Additional file 1, and the procedure was as follows. First, participating PCCs were matched based on their CNI by pairing 1:1 adjacent values of CNI, after ranking them from highest to lowest. The difference between adjacent values was judged in most cases as very small (i.e., lower than 2%). Thereafter, randomization was conducted within each matched pair. In one case, three PCCs were matched together due to the uneven number of PCCs. Out of the 31 PCCs included in the first step of recruitment, 16 were randomly allocated to HDI and 15to OS. The additional PCC that was included in a second step was randomly allocated to OS. Thus, a total of 16 PCCs were allocated to HDI and 16 to OS.

The statistician (APdL) who performed the randomization, and who will perform the outcome analysis, was blinded to the meaning of the computer-generated random numbers which determined the allocation of clusters to either HDI or OS. However, given the open-label and pragmatic design, participants and outcome assessors will not be blinded. As such, emergency blinding will not occur in this trial.

### Recruitment of participants, eligibility criteria, and informed consent

Patients aged 50–59 years old who are enlisted at the participating PCCs will be considered eligible for invitation to participate in the trial. Each of the PCCs will be asked to invite and enroll patients until they have reached *n* = 100 participants. For PCCs randomized to HDI, a healthcare professional (registered nurse or general practitioner) from the PCC will send a letter by mail to eligible patients, providing brief information about the intervention and a scheduled date and time for a phone call. During the phone call, the healthcare professional will provide more information about the intervention and the potential participant will have the opportunity to ask questions. Previous research indicates that this approach (information by ordinary mail followed by a phone call) could increase attendance rates compared to a mail-only invitation [26, 27]. If the patient expresses an interest in participating, an appointment for the screening and the health dialogue will be scheduled. Based on all enlisted patients, the healthcare professional will invite patients consecutively based on alphabetical order of their surnames. Patients choosing to participate will provide written informed consent and have the possibility to withdraw their participation at any time without the need to state a reason (Additional file 2). As part of the participant information and informed consent, participants are made aware that the trial is designed to evaluate how well HDI and OS can detect biological risk factors (surrogate endpoints) for T2D and CVD (target outcomes) and whether they lead to reduced risk of T2D and CVD in the long-term.

**Fig. 2:**
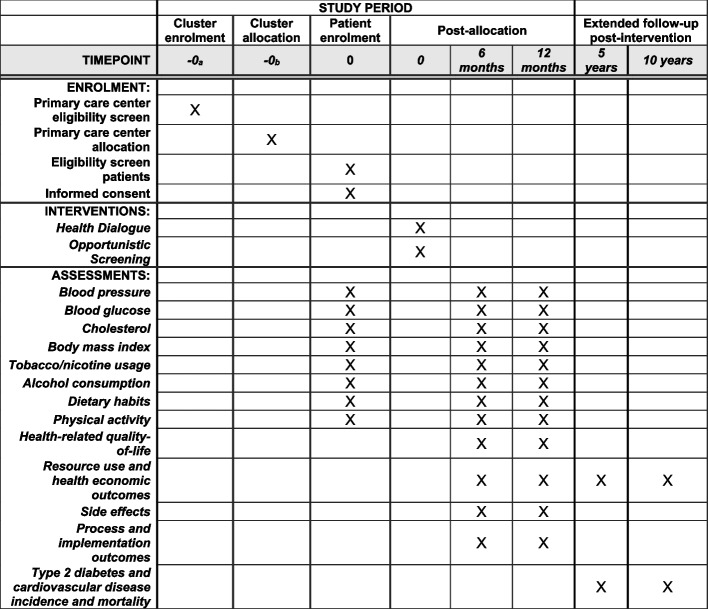
SPIRIT figure for schedule of enrollment, intervention, and assessment

For PCCs randomized to OS, eligible patients will be identified upon scheduling an appointment at their PCC for any reason, except for those whose scheduled appointments are related to hypertension, T2D, and CVD, or acute events requiring immediate care or immediate referral. Upon scheduling the appointment, the healthcare professional (assistant nurse, registered nurse, or general practitioner) will provide brief information about the trial. Next, once the patient arrives for their visit, they will again be given information about the trial. If agreeing to participate, they will provide written informed consent and have the possibility to withdraw their participation at any time without the need to state a reason (Additional file 3). Figure 2 shows the timeline of the study, including timepoints for enrollment, allocation, and assessments, as per SPIRIT guidance.

Additionally, from each of the participating PCCs, we will invite healthcare professionals for an assessment of process and implementation outcomes. For PCCs randomized to HDI, we will invite the healthcare professional that has been responsible for conducting the health dialogue and possibly also the manager. For PCCs randomized to OS, we will invite all healthcare professionals who have been implementing the intervention. Those agreeing to participate will provide written informed consent and have the possibility to withdraw their participation at any time without the need to state a reason (Additional file 4), after which they will respond to a questionnaire. A selection of healthcare professionals will be invited to participate in a semi-structured interview, as described in later sections.

### Intervention descriptions

#### Health dialogue intervention (HDI)

Patients consenting to participate in the trial, and enlisted at a PCC randomized to HDI, will receive an intervention following the Swedish prevention program for lifestyle-focused health dialogues [15]. The program includes a systematic screening assessment which forms a risk profile (see below). Based on the results of the assessment and the risk profile, participants will be offered an individually oriented lifestyle-focused health dialogue. The program has been developed in Swedish primary care since the mid-1980s [13, 14], and is briefly mentioned in the National Guidelines for Prevention and Treatment of Unhealthy Lifestyle Habits, developed by the Swedish National Board of Health and Welfare [7]. The model is currently used in 13 of the 21 regions in Sweden, with an additional five regions running pilot programs or launching the program shortly. In short, the program aims to map risk factors for T2D and CVD among all individuals in certain age groups, and to provide knowledge and subsequent guidance and support behavior change with respect to lifestyle habits, including subsequent follow-ups, with the primary goal of preventing T2D and CVD [15].

The intervention will be conducted according to the following procedure. First, ahead of the scheduled health dialogue, participants will be asked to fill out a health survey. For participants with language barriers, it will be possible to fill out the survey with the assistance from an interpreter in their native language in conjunction with the forthcoming health dialogue. The survey assesses various aspects of health status and lifestyle habits, including medical history, medications, general health, mental health, heredity, living situation, quality-of-life, alcohol consumption, dietary habits, tobacco and/or nicotine use, and physical activity. Second, participants will be instructed to leave blood samples no more than 7 days prior to the scheduled health dialogue, where cholesterol levels and glucose levels are measured. Finally, the participants will arrive at their PCC for the scheduled health dialogue. The appointment will begin with an assessment of body weight, height, waist-to-hip ratio, and blood pressure. These data will then be incorporated, together with the blood sampling results and the survey data, into a risk profile assessment tool providing a visual result of the participant’s risk profile, known as the “Health Curve.” The risk profile displays a gradient in CVD risk ranging from green (low risk), yellow, to red (high risk) [14, 28]. Finally, the healthcare professional will use motivational interviewing techniques to perform an individually oriented, lifestyle-focused health dialogue guided by the risk profile. In total, the appointment is scheduled to take about 75–90 min including preparation time, the health dialogue, and administration. The actual dialogue is expected to take about 60 min, and for participants in need of an interpreter, an additional 30 min is expected. Notwithstanding the personalized health dialogue, detected risk factors for T2D and CVD (e.g., hypertension) will also be managed in accordance with existing care programs and clinical practice guidelines (https://viss.nu/). There are no criteria for discontinuing the intervention. There is no concomitant care that is prohibited during the trial.

#### Opportunistic screening (OS)

**Fig. 3:**
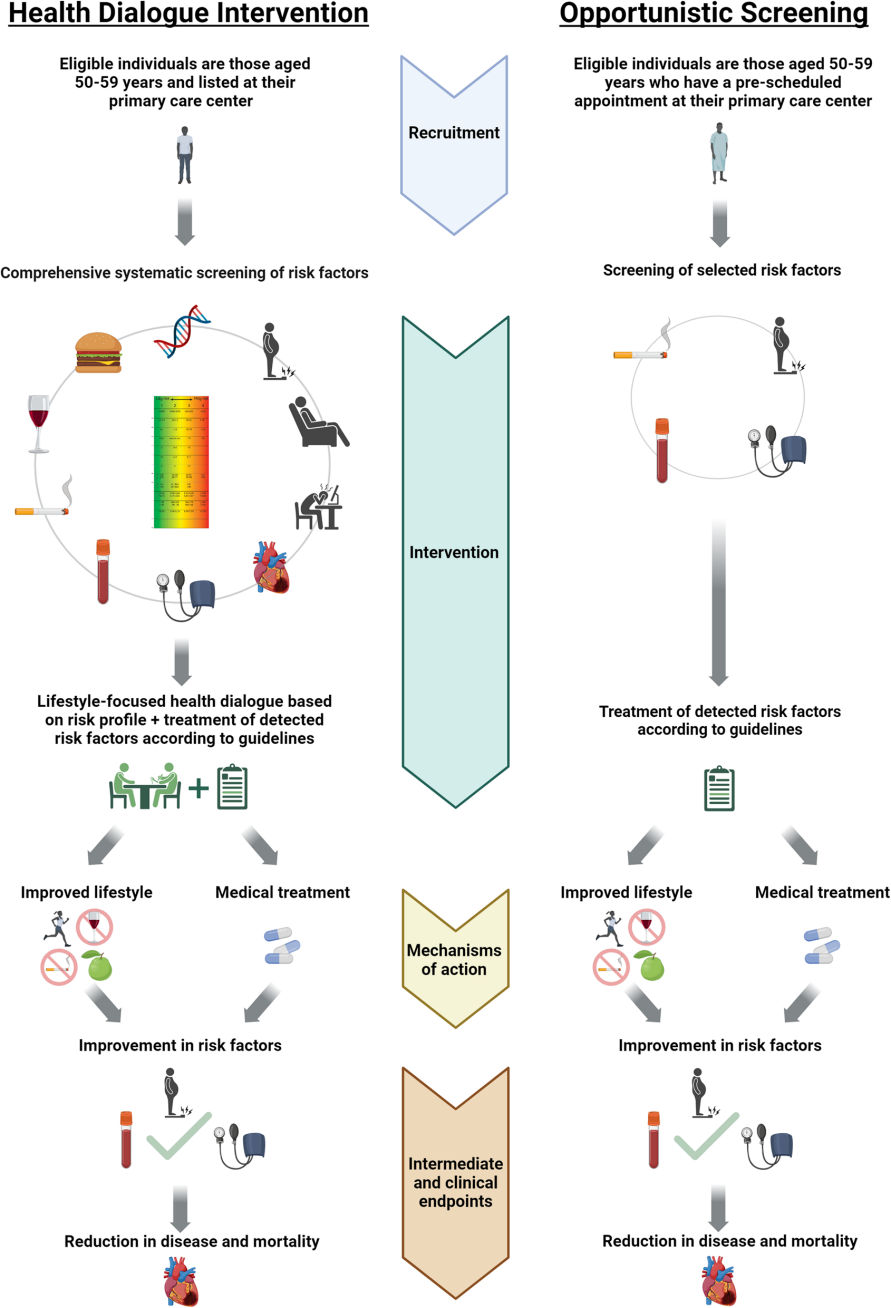
Visual illustration of the core components of the Health Dialogue Intervention and the Opportunistic Screening. Created with BioRender.com

Patients consenting to participate in the trial, and enlisted at a PCC randomized to OS, will receive an intervention including screening for a smaller set of selected risk factors. The intervention will be conducted as follows. In conjunction with the participant’s scheduled appointment at the PCC, a screening is conducted by the healthcare professional with whom the patient has the appointment. The screening includes measurement of blood pressure, body weight, and height, and the patient is asked about tobacco usage. In total, the appointment is scheduled to take about 15 min. Thereafter, the patient is instructed to provide blood samples within 7 days, where cholesterol levels and glucose levels will be measured. Detected risk factors for T2D and CVD will be managed according to the existing care programs and clinical practice guidelines (https://viss.nu/), which should always include lifestyle advice as the first intervention. However, unlike the HDI, participants will not be offered an individually oriented health dialogue. There are no criteria for discontinuing the intervention and there is no concomitant care that is prohibited during the trial. Figure 3 presents a visual illustration of the core components of HDI and OS.

### Implementation strategies

The implementation strategies that will be used to support implementation of the two interventions are labeled according to the Expert Recommendations for Implementing Change compilation of implementation strategies [29].

#### Educational meetings and educational materials

Prior to the start of the trial, all healthcare professionals providing the HDI intervention will complete a formal education in lifestyle-focused health dialogues as per the Swedish prevention program (1 day) and healthy lifestyle behaviors (1–2 days). Additionally, those not previously trained in motivational interviewing will complete a 2-day education in the method. For healthcare professionals providing the OS intervention, a short educational meeting will be provided to all healthcare professionals. The meeting will describe the purpose of the screening, how to conduct the screening, and measures to take when risk factors are detected, which includes treatment according to the existing care programs and clinical practice guidelines (https://viss.nu/). Educational materials detailing how the interventions should be delivered will be developed and distributed to the healthcare professionals in both arms prior to the trial.

#### Ongoing consultation

In both arms, ongoing consultation will be provided on an as-needed basis to continuously support the healthcare professionals delivering the interventions and help them solve potential problems. The consultation will be provided either at the PCC or online by a member from the project team.

### Outcomes and measures

This trial will assess a wide range of outcomes which can be grouped according to the following domains: biological risk factors, lifestyle habits, process and implementation, unintended side effects, short-term health outcomes, and long-term health outcomes and resource use.

### Biological risk factors

Biological risk factors, used as surrogate outcomes for the target outcomes CVD and T2D, will be assessed at baseline and at 6 months and 12 months post intervention. These include systolic blood pressure (primary outcome), body mass index, fasting blood glucose, total cholesterol, high-density lipoprotein cholesterol, and low-density lipoprotein cholesterol. The main reason for using systolic blood pressure as a primary outcome rather than the target outcomes was practical, as the internal funding for the trial was limited to 2025, for which it would not have been possible to determine the effect of the intervention on the target outcomes (i.e., CVD and T2D incidence). Additionally, the use of surrogate endpoints was justified based on the large-scale evidence on their implications in CVD and T2D [1, 30, 31]. Blood pressure (mmHg) will be measured in a seated position using digital devices. Two measurements will be made with a one-minute rest in-between. The mean value of the two measurements will be recorded. Body weight will be measured using digital scales and height using stadiometers, after which the body mass index will be calculated as the weight in kilograms over the height in meters squared. Blood samples (mmol/l) will be drawn and analyzed according to routine practice at accredited laboratories associated with each of the participating PCCs. All participants are instructed to leave blood samples in a fasted (≥ 12 h) state.

### Lifestyle habits

Dietary habits, tobacco/nicotine use, alcohol consumption, and physical activity will be assessed at baseline and at 6 months and 12 months post intervention through self-report using questions recommended by the National Board of Health and Welfare [32]. The specific questions and response categories are outlined in Additional file 5.

### Short-term health outcomes

The HRQoL will be assessed at baseline and at 6 months and 12 months post intervention using the EQ-5D instrument [33]. The EQ-5D is a generic patient-reported outcome measure of HRQoL across five dimensions (mobility, self-care, usual activities, pain/discomfort, anxiety/depression), each with five response options. Using predetermined value sets, an index value can be derived from the responses in the five dimensions. In this study, a Swedish value set recommended by EuroQol will be used [34]. For generalizability of results, index values based on additional value sets will also be calculated, in line with updated guidelines at the time of conducting the study.

### Long-term health outcomes and resource use

To examine the effect on long-term health outcomes, cases of incident T2D and CVD (ischemic heart disease and stroke) and deaths due to T2D and CVD will be collected during 5 and 10 years of follow-up from regional and nationwide health registers, using International Classification of Diseases, 10th revision (ICD-10) codes E11 for T2D and I00-I99 for CVD. The registers that will be used are the National Patient Register, the National Cause of Death Register, the National Diabetes Register, and the Stockholm Regional Healthcare Data Warehouse. The National Patient Register includes data on all inpatient care in Sweden with 100% coverage since 1987, and since 2001, it also includes data on specialized outpatient care [35]. The Cause of Death Register covers data on all deaths in Sweden since 1952 [36]. Both of these registers are managed by the National Board of Health and Welfare. The National Diabetes Register was initiated in 1996, has 87% coverage, and includes data on patients with a diagnosis of diabetes, regardless of the care provider, thus including also primary data in contrast to the National Patient Register [37, 38]. This register is managed by Register Centre, Västra Götaland, Sweden. The Stockholm Regional Healthcare Data Warehouse (VAL) is a regional database including data on diagnoses and healthcare utilization from inpatient, specialized outpatient, and primary care for all people living in Stockholm Region since 1997 [39]. The coverage is complete since 2013 [37]. Additionally, information on prescribed medications for T2D and CVD (at baseline and during follow-up) will be collected from the Prescribed Drug Register using Anatomical Therapeutic Chemical codes A10, C02-C03, C07-C10. The Prescribed Drug Register is managed by the National Board of Health and Welfare and includes data on all prescribed medications that are collected at Swedish pharmacies since July 2005 [40]. Cost data will be collected from regional pricelists, the Cost Per Patient database, held at the Swedish Association of Local Authorities and Regions, and the national pricelist of reimbursed medications, held at the Dental and Pharmaceutical Benefits Agency.

### Process and implementation outcomes

Process and implementation outcomes will be assessed 6 months post intervention according to Proctor et al.’s taxonomy [41]. Adoption will be measured as the number of invited PCCs that agree to participate in the study. Healthcare professionals’ perceptions of how acceptable, appropriate, and feasible the interventions are to implement within the context of the PCCs will be assessed using the Acceptability of Intervention Measure, the Intervention Appropriateness Measure, and the Feasibility of Intervention Measure [42]. The measures consist of four items each and responses are reported on a 5-point Likert scale ranging from “completely disagree” to “completely agree.” Fidelity will be operationalized as adherence to the original intervention protocols and assessed using data from electronic medical records. For the HDI, we will report to what extent (proportion) all parameters of the screening have been assessed and whether the following health dialogue has been conducted, as based on documentation in the patients’ electronic medical records. For the OS, we will assess to what extent (proportion) all parameters of the screening have been assessed and to what extent that identification of an elevated risk factor has resulted in measures taken according to existing clinical practice guidelines. (For example, what proportion of participants who report daily smoking are given brief advice about smoking cessation and asked whether they want a referral to a qualified tobacco cessation advisor.) Penetration will be assessed in terms of the proportion (in both arms) of patients choosing to participate among those invited (attendance rate), and the reasons for declining participation among non-participants. We will also report (in both arms) the representativeness of participants as compared to the total eligible population based on socioeconomic characteristics. Participants data on sex, level of education, occupation, annual income, and birth country will be compared to that of the total eligible population, using data from the Longitudinal Integration Database for Health Insurance and Labour Market Studies (LISA) [43]. LISA is managed by Statistics Sweden (the agency of government statistics), which has a 100% coverage for all Swedish citizens aged > 16 years since 1990 and for all aged > 15 years since 2010. In Stockholm Region, the data from LISA is already integrated with the VAL database and used routinely. Additionally, we will assess case-finding rates (in both arms) defined as the proportion of participants where elevated levels of T2D and CVD risk factors are detected for the first time, e.g., the proportion of participants with prediabetes defined as a fasting blood glucose of 6.1–6.9 mmol/l and hypertension defined as blood pressure of ≥ 140/90 mmHg. Finally, semi-structured interviews will be conducted with one healthcare professional and potentially one manager from each participating PCC, with the aim of exploring barriers to and facilitators for implementation of the interventions. The interviews will be audio-recorded, transcribed verbatim, and analyzed using deductive content analysis [44]. The Consolidated Framework for Implementation Research will be used to guide the analysis [45]. Costs related to implementing and running HDI and OS will be analyzed using a bottoms-up, micro-costing approach. This includes staff training, time spent delivering the interventions, necessary materials, IT-infrastructure, etc.

#### Time needed to treat (TNT)

With respect to implementation, we will also estimate the TNT for the interventions. The TNT is a new method designed to consider clinician’s time as a finite resource, with the aim of facilitating for guideline committees who develop clinical practice guidelines. The TNT can be expressed in three different ways: (I) the clinician time needed to improve the outcome for one person (TNT_NNT_), (II) the clinician time needed to provide the intervention for all eligible in a population (absolute TNT), and (III) the proportion of the total clinician time available for patient care needed to implement the intervention for everyone eligible (relative TNT). More detailed information on the TNT method and its assumptions is available elsewhere [46].

### Unintended side effects

Unintended side effects of the interventions experienced by study participants will be assessed through self-report at baseline and at 6 months and 12 months post intervention. Participants will be asked to rate their degree of perceived stress during the past 12 months on a scale from 0 to 50, ranging from “no stress” to “very high level of stress.” They will also respond to a question which reads “Have you experienced any negative consequences as a result of your participation in the trial?”, with two fixed response categories (“yes” or “no”). Those responding “yes” will then be asked to provide an open answer, specifying the negative consequences. Additionally, the collection of data on the target outcomes (CVD and T2D incidence) through extended observational follow-up will provide additional evidence regarding whether any short-term benefits in surrogate markers translate into reduced risk of the target outcomes, of importance from a risk–benefit balance discussion.

### Statistical analysis

Baseline characteristics in the two arms will be described by means of typical summary measures e.g., means, medians, frequencies, proportions, standard deviations, quartiles, and counts. Characteristics of dropouts will be explored in comparison to non-dropouts.

The core tool of outcome analysis will be a hierarchical model consisting of two levels, individuals and PCCs. The primary model for analyzing changes in systolic blood pressure (primary outcome), other biological risk factors, lifestyle habits, and HRQoL over 6 and 12 months will be a mixed-effects generalized linear model (GLM) to capture variability between and within PCCs. Following the GLM framework, the assumptions concerning the underlying probability distribution and the link function (the way the expected value of the outcome is related to a set of independent variables and their associated effects) will differ between continuous, binary, categorical, and count outcomes. In these models, the treatment effect (HDI versus OS) will be assessed using a fixed effect represented by a dummy variable at PCC level, specifying the type of treatment (HDI/OS). Together, this enables the estimation of the average treatment effect, while accounting for the hierarchical structure of the data. We will estimate unadjusted effects and effects adjusted both/either at the individual level and PCC level, e.g., adjusted for demographic characteristics, socioeconomic status, CNI level, and baseline values of the referred outcome. The analyses will be conducted on an intention-to-treat basis and will be carried out using the *glmer* function of the *lme4* package under the R platform [47]. Potential effect modification will be explored through subgroup analyses and interaction analyses considering sex, birth country, and socioeconomic characteristics. A sensitivity analysis will be conducted to assess whether the individual randomization of a single PCC introduced bias. Thus, we will perform analyzes both including and excluding this PCC. The occurrence of missing data will be dealt with accordingly.

For the assessment of T2D and CVD incidence and mortality during the extended 5 and 10 years of follow-up, we will calculate hazard ratios using a mixed-effect Cox regression model and packages *survival* and *coxme* under the R platform [48–50]. We will also calculate the number needed to treat (NNT).

#### Health economic evaluation

For the short-term health economic evaluation, a healthcare costing perspective will be used, including utilization of healthcare in primary, specialized outpatient, and inpatient care as well as medication. Data from the VAL database will be used for this analysis. Total health resource use and associated costs in each arm will be accumulated and analyzed over the 12-month follow-up. Net costs, which is the difference between intervention costs and total health resource use of participants, will thereafter be estimated for both arms. Total QALYs at 6 and 12 months in each arm will be calculated by using the area under the curve method [51]. Incremental differences in QALYs and costs between participants in the HDI and OS arm at 6 and 12 months will be assessed using GLM models with gamma distribution for cost outcomes and gaussian for QALYs. Logistic regression analyses will be used for estimating between-group differences in persons with blood pressure target attained. Between-group differences in healthcare costs will be analyzed while controlling for baseline costs, while baseline utilities will be controlled for in analyses of accumulated QALYs between groups. The cost per QALY and per blood pressure target attained will be assessed using the incremental differences between the groups at 12 months.

For the long-term assessment of cost-effectiveness, total healthcare resource use and its associated costs will be accumulated and analyzed over the 5- and 10-year follow-up period, employing the same data source (VAL) and analytical framework (GLM) as for the short-term assessment. Intervention costs will also be used in this evaluation for the estimation of net costs. Incident cases of T2D and CVD will similarly be accumulated over the follow-up period, and incremental differences at 5 and 10 years will be assessed using logistical analyses. The total costs and health outcomes will be analyzed in a cost-consequence analysis. Furthermore, accumulated deaths due to T2D and CVD over the 5- and 10-years follow-up will be used to assess incremental life years gained between the two groups, and this outcome measure will consecutively be used in a cost-effectiveness analysis estimating the cost per life years gained. Probabilistic sensitivity analyses will be employed throughout the short- and long-term analyses to account for parameter uncertainty.

### Data collection and data management

All clinical assessments at baseline and at 6 months and 12 months post intervention are performed by the healthcare professionals involved in the trial at each of the PCCs, except for the blood sampling which, as described earlier, will be performed by staff at the accredited laboratories associated with the PCCs. To meet the diversity of language among participants, the questionnaires will be available in Swedish, English, Arabic, Turkish, Somali, Tigrinya, Russian, Spanish, and Dari. Additionally, for participants belonging to PCCs randomized to HDI, and who have language barriers, it will be possible to have assistance from an interpreter during the health dialogue.

Because the clinical data will be collected as part of the pilot program at the PCCs, it will be stored in the patients’ electronic medical records as per routine procedures. The data from the patients’ electronic medical records will be integrated with the register-based data, as well as the manually entered survey data, and stored securely on servers at the Centre for Epidemiology and Community Medicine, Stockholm County Health Care Area, Region Stockholm, where only authorized researchers will have access. A member of the research team will perform range checks of the data. The physical consent forms will be stored securely at the PCCs according to standard safety procedures, and the information will also be entered manually into a database and stored securely on servers at the Centre for Epidemiology and Community Medicine, Stockholm County Health Care Area, Region Stockholm.

### Data retention plan

Members of the research group will provide updates on the trial status to the healthcare professionals at the PCCs who participate in the trial. Moreover, the healthcare professionals will provide all study participants with a reminder of their scheduled assessment ahead of each assessment (baseline, 6-month follow-up, 12-month follow-up). Participants who miss their assessment will be given the possibility to reschedule. For the assessment of long-term health outcomes, loss to follow-up should be minimal thanks to the use of high-coverage registers.

### Confidentiality of data

All data will be handled in accordance with Swedish law and the EU General Data Protection Regulation. The data will be stored securely on servers at the Centre for Epidemiology and Community Medicine, Stockholm County Health Care Area, Region Stockholm, where only authorized researchers will have access. The data will be pseudo-anonymized and analyzed and presented only at an aggregated level; hence, data for specific individuals will not be presented. The data protection officer of Stockholm County Health Care Area is also the data protection officer of this study.

### Dissemination of results

The results will be presented in written format through reports issued by the Centre for Epidemiology and Community Medicine, Region Stockholm, as well as through scientific articles in peer-reviewed journals. Verbal dissemination will occur through presentations at internal and external meetings as well as through conferences.

## Discussion

The DETECT trial aims to evaluate the real-world effectiveness, cost-effectiveness, and implementation of a systematic screening for T2D and CVD risk factors followed by an individually oriented, lifestyle-focused, health dialogue, as compared to opportunistic screening, in primary care in socioeconomically disadvantaged areas in the largest region of Sweden. Notwithstanding the current use of the Swedish prevention program involving targeted health dialogues in most of the regions of Sweden, this trial will likely be one of the most ambitious attempts to assess its effectiveness, cost-effectiveness, and implementation. The overall pragmatic approach and assessment of a rich set of diverse outcomes has the potential to contribute with valuable information that could support evidence-based policymaking in the region and underpin forthcoming political decisions regarding whether scale-up of the pilot program across PCCs is justified.

This trial will have limitations that should be considered. First, although the pilot program underlying this trial employs an overall pragmatic approach, certain measures had to be taken that may interfere with what would be considered pragmatic. For instance, some implementation strategies had to be applied. This was deemed necessary given that even though health dialogues are used across many regions in Sweden, and opportunistic screening may be used occasionally, neither of these are part of the systematic core activities of Swedish primary care. As such, we believe that implementation strategies must also be considered in any future scale-up of the interventions in clinical practice. Second, we acknowledge a risk of selection bias due to post-randomization recruitment of participants that are not blinded to treatment allocation [52]. Various mitigation strategies have been proposed to reduce this bias, such as using blinded independent recruiters and providing both arms with similar trial information and consent forms [52]. While unable to use a blinded independent recruiter in our trial, we kept trial information and consent forms similar for HDI and OS. This information is published together with this protocol, to increase transparency and aid in interpretation of trial results [52]. Nevertheless, some bias will likely remain, which could affect (in an unpredictable direction) the internal validity of the head-to-head comparison of the two interventions. Consequently, to tackle imbalances in characteristics (at both cluster and individual level) between arms, the analyses will employ covariate adjustment, which has been deemed particularly important in trials where participants are unblinded and recruited post-randomization [52]. Third, this trial will not include a care as usual care-arm. If one would assume that OS may be more effective than usual care, while recognizing that OS is the comparator in this trial, this could in theory mean that any potential benefits from HDI may become masked due to insufficient exposure contrast. However, a previous meta-analysis found that compared to OS, even systematic screening interventions of lower intensity than our HDI intervention may have favorable effects on some risk factors, including a 3-mmHg reduction in systolic blood pressure [9]. Moreover, a head-to-head comparison of HDI and OS will be relevant for clinical practice given that OS is an approach that is sometimes used by clinicians, although not systematically. Fourth, we made several sample size calculations with varying assumptions to ensure sufficient statistical power under different scenarios that may occur due to attrition. Although these calculations suggested that we should have sufficient statistical power even with many dropouts, unexpected situations may emerge that are beyond our control. For example, PCCs facing high staff turnover and/or saving requirements could be reorganized and shift resources towards core activities, away from trial participation.

There are also potential strengths with this trial. One is the large-scale, real-world evaluation of preventive approaches that are commonly used in clinical practice despite their inconclusive evidence. Another one is the target setting (i.e., areas with a lower socioeconomic status and a high concentration of foreign-born citizens) for which the current body of evidence is limited. Moreover, there will be an assessment of a vast number of outcomes across multiple domains that are relevant to a wide number of groups. This includes assessment of multiple health-related outcomes on a patient-level, documentation of unintended side effects on both patient- and caregiver-level, and several outcomes relevant for clinicians, healthcare directors, politicians, and policymakers, including resource use, cost-effectiveness, and process and implementation outcomes. Indeed, data on potential harms experienced by both study participants and healthcare professionals, as well as cost-effectiveness of the intervention, are particularly important features given that they have rarely been assessed in previous trials [9]. Collectively, this multifaceted and comprehensive assessment of lifestyle-focused health dialogues and opportunistic screening in primary care in socioeconomically disadvantaged areas has the potential to add valuable knowledge that could support evidence-based policymaking.

### Trial status

The trial was prospectively registered with ClinicalTrials.gov (NCT06067178) on September 27, 2023. This was before the recruitment of the first study participant, which occurred on December 1, 2023. Participant recruitment is planned to be completed in December 2024, and data collection (except for long-term health outcomes) is planned to be completed in December 2025. Potential changes to the protocol will be handled and communicated as follows. First, the principal investigator will discuss the proposed changes together with the steering committee; after depending on the severity of the proposed changes, the internal funder will also be consulted. Following approval of the changes, the principal investigator will notify the staff at the PCCs of the changes. Finally, the protocol will be modified according to the changes and updated in the ClinicalTrials registry. The protocol version of the study protocol at the time of submission to *Trials* was 4.0 (last edited 2024 01 30).

## Supplementary Information

Supplementary Material 1.

Supplementary Material 2.

Supplementary Material 3.

Supplementary Material 4.

Supplementary Material 5.

Supplementary Material 6.

## Data Availability

No data is included in this protocol. The data that will be collected in this trial cannot be shared or made publicly available due to Swedish law and regulations. Statistical code underlying the forthcoming analysis in the reporting of trial results will be made available.

## Abbreviations

CNI: Care Need Index
CONSORT: Consolidated Standards of Reporting Trials
CVD: Cardiovascular disease
DETECT: Health Dialogue intervention versus opportunistic screening in primary Care for Type 2 diabetes and cardiovascular disease prevention in settings with low socioeconomic status
EQ-5D: EuroQoL 5D
HDI: Health Dialogue Intervention
HRQoL: Health-related quality-of-life
GLM: Generalized linear model
ICD: International Classification of Diseases
LISA: Longitudinal Integration Database for Health Insurance and Labour Market Studies
NNT: Number needed to treat
OS: Opportunistic Screening
PCC: Primary care center
QALY: Quality-adjusted life year
SED-GIH: GIH stationary single-item question
SPIRIT: Standard Protocol Items: Recommendations for Interventional Trials
TNT: Time needed to treat
T2D: Type 2 diabetes
VAL: Stockholm Regional Healthcare Data Warehouse (VAL)
