# Supplementary material for: Health dialogue intervention versus opportunistic screening in primary care for type 2 diabetes and cardiovascular disease prevention in settings with low socioeconomic status (DETECT): study protocol for a pragmatic cluster-randomized trial
Source: Trials. 2024 Oct 12;25:672. doi: 10.1186/s13063-024-08533-8 (PMC11470558; doi:10.1186/s13063-024-08533-8)
Supplement: Supplementary file 2 — Supplementary Material 2. [file 13063_2024_8533_MOESM2_ESM.pdf]

Region Stockholm

Evaluation of targeted healthcare conversations and screening in primary care for prevention of cardiovascular disease and type 2 diabetes

We want to ask you if you would like to participate in the study *Evaluation of targeted healthcare conversations and screening in primary care for the prevention of cardiovascular disease and type 2 diabetes*. In this document, you will find information about the study and what it entails to participate.

What kind of study is it and why do we want you to participate?
The purpose of the study is to investigate how the primary health care can best detect risk factors for cardiovascular disease and type 2 diabetes. Cardiovascular disease and type 2 diabetes are among the most common diseases in Sweden. Both are lifestyle diseases that can often be prevented or slowed down by early detection and treatment of risk factors.

Therefore, a study is currently ongoing with targeted healthcare conversations and screening at your healthcare centre, with the aim of increasing the detection of risk factors in the population. You are one of those who have taken part in a healthcare conversation and you are therefore asked if you would like to participate in an evaluation of these methods. The purpose of the evaluation is to investigate how well the methods lead to the detection of risk factors and whether they prevent disease in the long term.

The study is being conducted by the Centre for Epidemiology and Community Medicine (CES), which is part of Region Stockholm. CES is also the entity responsible for the research. The application has been approved by the Swedish Ethical Review Authority. The case number for the review at the Swedish Ethical Review Authority is 2023-03001-01.

How is the study conducted?
If you agree to participate in the study, CES will receive your responses to the completed questionnaire in connection with the healthcare conversation. We will also receive information about your blood pressure, results from blood tests and body measurements, as well as any follow-up healthcare visits linked to the healthcare conversation from your patient records. In order to gain a more comprehensive understanding of cardiovascular disease and type 2 diabetes, and how targeted healthcare conversations and screening affect these diseases, this data is supplemented with information about your health and background from registries at Region Stockholm, Region Västra Götaland, Statistics Sweden and the National Board of Health and Welfare. The data concerns family, migration, domicile, residential area, employment, education, diagnoses, health, healthcare events and medicine use. In order to be able to take any heredity into consideration, we also collect information about cardiovascular disease and diabetes from your closest relatives.

What will happen to my data?
The study will collect and record information about you. It will not be evident what

Centre for Epidemiology and Community Medicine
Box 45436
104 31 Stockholm
Telephone switchboard:08-123 400 00
Email: ces.slso@sll.se
Org.no.: 232100-0016
www.folkhalsoguiden.se

information comes from you when the results of the study are presented. The analyses are made with pseudonymised data, which means that all identities have been replaced with sequential numbers. The code key is stored separately from your data. We only use the data required for the analysis in question. Since research is considered to be in the public interest, CES has the right, in accordance with Swedish jurisdiction, to manage and store the personal data you agree to share with us. The data will be archived for at least ten years. We manage your data in accordance with Swedish jurisdiction and the EU's General Data Protection Regulation (GDPR). All collected data is stored on an access-protected file server at CES and will only be accessible to authorised researchers with personal login codes. The information will be treated confidentially and no information that can be traced back to specific persons will be disclosed. In this way, we protect your privacy. You have the right to access the data about you that is processed in the study, free of charge, in accordance with EU's General Data Protection Regulation. You can do this by submitting a written request to the responsible researcher (see contact details below). Should any information be incorrect, you have the right to have the information corrected or deleted. If you have any comments regarding CES's processing of your personal data, you can contact SLSO's Data Protection Officer: gdpr@slso@regionstockholm.se. If you are not satisfied with SLSO's response, you can contact the Swedish Authority for Privacy Protection: imy@imy.se

How do I get information about the results of the study?
The results of the study will be used to investigate how primary care can become better at detecting people at risk of developing cardiovascular disease and type 2 diabetes. The results will also be used to follow up on preventive treatment and support to change lifestyles. The results will be presented in a report and in scientific articles. If you would like to take part of the research results, please register your interest by email to the responsible researcher (please see contact details below).

Insurance and compensation
Participants in the study are insured by regular patient insurance. You will not receive any financial compensation for participating.

Participation is voluntary
Participation is voluntary, and you can withdraw your participation at any time. You can participate in the healthcare conversation without participating in research. If you choose not to participate or wish to withdraw your participation, you do not need to state a reason. It will also not affect your future care. Should you wish to withdraw your participation, please contact the person responsible for the study (see below).

Dealing with unexpected findings
Discovered health problems are handled by the healthcare centre/primary care centre according to usual routines.

Responsible for the study and contact details

Hanna Augustsson, Principal Investigator
Unit for Implementation and Evaluation, Centre for Epidemiology and Community Medicine (CES)
Email address: Hanna.augustsson@regionstockholm.se
Telephone number: 08-123 371 26

4 (5)

Consent to participate in the study *Evaluation of targeted healthcare conversations and screening in primary care for the prevention of cardiovascular disease and type 2 diabetes*

To confirm that you have received this information, we require a written signature on this document.

- I consent to participate in the study *Evaluation of targeted healthcare conversations and screening in primary care for the prevention of cardiovascular disease and type 2 diabetes*
- I have read and understood information about the study and had the opportunity to ask questions
- I consent to the processing of my information and personal data in the manner described in the section regarding processing of personal data

Place and date	Signature
	Name in print
