# Supplementary material for: Health dialogue intervention versus opportunistic screening in primary care for type 2 diabetes and cardiovascular disease prevention in settings with low socioeconomic status (DETECT): study protocol for a pragmatic cluster-randomized trial
Source: Trials. 2024 Oct 12;25:672. doi: 10.1186/s13063-024-08533-8 (PMC11470558; doi:10.1186/s13063-024-08533-8)
Supplement: Supplementary file 4 — Supplementary Material 4. [file 13063_2024_8533_MOESM4_ESM.pdf]

Region Stockholm

Evaluation of targeted healthcare conversaions and screening in primary care for prevention of cardiovascular disease and type 2 diabetes

We want to ask you, in the role as a healthcare professional, if you would like to participate in the study *Evaluation of targeted healthcare conversations and screening in primary care for the prevention of cardiovascular disease and type 2 diabetes*. In this document, you will find information about the study and what it entails to participate.

What kind of study is it and why do we want you to participate?

The study evaluates targeted healthcare conversations and opportunistic screening in primary care for prevention of cardiovascular disease and type 2 diabetes. The study evaluates effectiveness, costs, and implementation of the interventions. Your primary care center is participating in the study, and you belong to the group of healthcare professionals who have been working in the study. You are therefore asked to participate.

Questionnaire: The purpose of the questionnaire is to explore your experiences of working with the intervention. We want to learn your thoughts about your perceived barriers and facilitators of implementing the intervention.

Interview: The purpose of the interview is to further explore your experiences of working with the intervention. We want to learn your thoughts about your perceived barriers and facilitators of implementing the intervention.

The study is being conducted by the Centre for Epidemiology and Community Medicine (CES), which is part of Region Stockholm. CES is also the entity responsible for the research. The application has been approved by the Swedish Ethical Review Authority. The case number for the review at the Swedish Ethical Review Authority is 2023-03001-01.

How is the study conducted?

Questionnaire: The questionnaire includes questions about appropriateness and feasibility of implementing the intervention at your primary care center. There are no right or wrong answers, rather, we are interested in your experiences and thoughts. The questionnaire takes about 10 minutes to complete.

Interview: During the interview you will respond to questions regarding your experiences of implementing the intervention. The interview is led by a member of the research group, who will ask questions about what barriers and facilitators that you identified when implementing the intervention. There are no right or wrong

answers. The purpose of the interview is to understand your experiences and thoughts. The purpose is not to evaluate you or your work-related performance. The interview takes about 30-45 minutes and will be recorded with your consent. Next, the interview will be transcribed and analyzed by the research group.

Potential risks from participating in the study
Your participating does not include risks. The information collected from the questionnaire/interview will be stored in a pseudonymized form that does not reveal your identity. Your employer will not have access to this information.

What will happen to my data?
Your data will be handled so that unauthorized individuals cannot take part of it. Results from the study will be presented on group level and not on an individual level. The presentation of results on an overall, general level means that it will not be possible to know the answers from specific individuals. Your employer, boss, or colleagues will not have access to the data from specific individuals. No information that can be connected to specific individuals will be presented.

Since research is considered to be in the public interest, CES has the right, in accordance with Swedish jurisdiction, to manage and store the personal data you agree to share with us. The data will be archived for at least ten years. We manage your data in accordance with Swedish jurisdiction and the EU's General Data Protection Regulation (GDPR). All collected data is stored on an access-protected file server at CES and will only be accessible to authorised researchers with personal login codes. The information will be treated confidentially and no information that can be traced back to specific persons will be disclosed. In this way, we protect your privacy. You have the right to access the data about you that is processed in the study, free of charge, in accordance with EU's General Data Protection Regulation. You can do this by submitting a written request to the responsible researcher (see contact details below). Should any information be incorrect, you have the right to have the information corrected or deleted. If you have any comments regarding CES's processing of your personal data, you can contact SLSO's Data Protection Officer: gdpr@slso@regionstockholm.se. If you are not satisfied with SLSO's response, you can contact the Swedish Authority for Privacy Protection: imy@imy.se

How do I get information about the results of the study?
The results will be presented in a report and in scientific articles. If you would like to take part of the research results, please register your interest by email to the responsible researcher (please see contact details below).

Insurance and compensation
Participants in the study are insured by regular workplace insurance. You will not receive any financial compensation for participating.

Participation is voluntary

Participation is voluntary, and you can withdraw your participation at any time. If you choose not to participate or wish to withdraw your participation, you do not need to state a reason. Should you wish to withdraw your participation, please contact the person responsible for the study (see below).

Responsible for the study and contact details

Hanna Augustsson, Principal Investigator
Unit for Implementation and Evaluation, Centre for Epidemiology and Community Medicine (CES)
Email address: Hanna.augustsson@regionstockholm.se
Telephone number: 08-123 371 26

Consent to participate in the study *Evaluation of targeted healthcare conversations and screening in primary care for the prevention of cardiovascular disease and type 2 diabetes*

To confirm that you have received this information, we require a written signature on this document.

- I consent to participate in the study *Evaluation of targeted healthcare conversations and screening in primary care for the prevention of cardiovascular disease and type 2 diabetes*
- I have read and understood information about the study and had the opportunity to ask questions
- I consent to the processing of my information and personal data in the manner described in the section regarding processing of personal data

Place and date	Signature
	Name in print
