# Supplementary material for: Health dialogue intervention versus opportunistic screening in primary care for type 2 diabetes and cardiovascular disease prevention in settings with low socioeconomic status (DETECT): study protocol for a pragmatic cluster-randomized trial
Source: Trials. 2024 Oct 12;25:672. doi: 10.1186/s13063-024-08533-8 (PMC11470558; doi:10.1186/s13063-024-08533-8)
Supplement: Supplementary file 5 — Supplementary Material 5. [file 13063_2024_8533_MOESM5_ESM.pdf]

Description of questions for the assessment of lifestyle risk factors

Diet, tobacco/nicotine use, alcohol consumption, and physical activity will be assessed at baseline and at 6 months and 12 months post intervention through self-report using questions recommended by the National Board of Health and Welfare.(1) The specific questions follows below.

Diet

Four questions will be used to assess the frequency of consumption of the following categories: fish, vegetables, fruit and berries, snacks and sugar-sweetened beverages. Each of the questions has four fixed response categories, ranging from “never”, “< once per month”, “monthly”, to “weekly”, including specification of the frequency of consumption for the chosen category (e.g., 3 times weekly), except for the question on fish consumption which has four fixed categories, ranging from “never” to “weekly”.

Tobacco

Six questions will assess tobacco usage. The first question reads: “Do you currently use, or have you previously been using, any tobacco products (e.g., snus, cigarettes, cigar, pipe) or other nicotine products (e.g., waterpipe, e-cigarettes, nicotine gum), with two fixed response categories (“yes” or “no”). The second question reads “Do you currently use, or have you previously been using snus (including white snus)?”, with four fixed response categories, ranging from “no”, “I have quit snus”, “Yes, sometimes”, to “Yes, daily or close to daily”, including specification of the number of boxes used (per day for daily users and per month for those using sometimes). Third, participants responding current usage of snus will be promoted

with the questions which reads: “If you answered yes, which type of snus are you using?”, with three fixed response categories, ranging from “brown snus”, “white snus”, to “both white and brown snus”. The fourth question reads: “Do you currently smoke, or have you previously been smoking cigarettes?”, with four fixed categories, ranging from “no”, “I have quit smoking cigarettes” (including date of quitting), “Yes, sometimes”, to “Yes, daily or close to daily”, including specification of the number of cigarettes smoked (per day for daily smokers and per month for those smoking sometimes). The fifth question reads: “Do you currently smoke, or have you previously been smoking waterpipe?”, with four fixed response categories, ranging from “no”, “I have quit smoking waterpipe” (including date of quitting), “Yes, sometimes”, to “Yes, daily or close to daily”, including specification of the number of sessions (per day for daily smokers and per month for those smoking sometimes). The sixth question reads: “Are you using nicotine replacement products (e.g., nicotine gum or nicotine patches)?”, with two fixed response categories (“yes” or “no”).

Alcohol

Three questions will be used to assess alcohol consumption. The first reads “How often do you drink alcohol?” and has five fixed response categories ranging from “never, “once per month or less”, “2-4 times per month”, “2-3 times per week”, to “4 times per week or more often”. The second question reads: “How many units do you drink during an ordinary week?” and has an open answer. The third question reads: “How often do you as female drink four units or more, and you as male five units or more, on one single occasion?”, and has five fixed response categories ranging from “never”, “less than once per month”, “monthly”, “weekly”, to “daily or almost daily”, including specification of the frequency of consumption for the chosen category. Participants responding “never” to the first question will be prompted to

answer an alternative question which reads: “Have you previously drunk alcohol?” and has three fixed response categories ranging from “yes”, “no”, to “I have tried on some occasion”.

Physical activity and sedentary time

Physical activity will be assessed using two questions. The first question aims to capture physical exercise, and reads “During a regular week, how much time do you spend exercising on a level that makes you short winded, for example running, fitness class, or ball games?”. The respondent has six fixed response categories (expressed in minutes) to choose from, ranging from “no time”, “< 30”, “30–60”, “60–90”, “90–120”, to “> 120”. The second question aims to capture everyday non-exercise physical activity and reads “During a regular week, how much time are you physically active in ways that are not exercise, for example walks, bicycling, or gardening? Add together all activities lasting at least 10 min.” The respondent has seven fixed response categories (expressed in minutes) to choose from when answering, ranging from “no time”, “< 30”, “30–60”, “60–90”, “90–150”, “150–300”, to “> 300”. These questions have been validated against accelerometer-measured physical activity, showing moderate agreement of a similar magnitude as the frequently used International Physical Activity Questionnaire.(2)

The amount of daily sedentary time will be assessed through self-report using the GIH stationary single-item question (SED-GIH).(3) The question reads “How much time do you sit a normal day, excluding sleep?” and has seven fixed response categories (expressed in hours) to choose from, ranging from “virtually all day”, “13–15 h”, “10–12 h”, “7–9 h”, “4–6 h”, “1–3 h”, to “never”. In a validation study, SED-GIH was found to have strong agreement with other self-reported sedentary time questions, and moderate agreement with accelerometer-assessed sedentary behavior.(3)

REFERENCES

1. The Swedish National Board of Health and Welfare. Nationella riktlinjer för sjukdomsförebyggande metoder 2011, Indikatorer, Bilaga [Disease Prevention in the Swedish Healthcare System: Health situation, national guidelines and implementation, Indicators, Appendix]. Västerås, Sweden: The Swedish National Board of Health and Welfare, 2011.
2. Olsson SJG, Ekblom Ö, Andersson E, Börjesson M, Kallings LV. Categorical answer modes provide superior validity to open answers when asking for level of physical activity: A cross-sectional study. Scand J Public Healt. 2016;44(1):70-6.
3. Kallings LV, Olsson SJG, Ekblom Ö, Ekblom-Bak E, Börjesson M. The SED-GIH: A Single-Item Question for Assessment of Stationary Behavior-A Study of Concurrent and Convergent Validity. Int J Env Res Pub He. 2019;16(23).
