# Supplementary material for: Health dialogue intervention versus opportunistic screening in primary care for type 2 diabetes and cardiovascular disease prevention in settings with low socioeconomic status (DETECT): study protocol for a pragmatic cluster-randomized trial
Source: Trials. 2024 Oct 12;25:672. doi: 10.1186/s13063-024-08533-8 (PMC11470558; doi:10.1186/s13063-024-08533-8)
Supplement: Supplementary file 6 — Supplementary Material 6. [file 13063_2024_8533_MOESM6_ESM.docx]

The combined SPIRIT-Surrogate extension checklist

| **Section/item** | **Item No** | **SPIRIT-Surrogate items** | **Reported on page No** |
| --- | --- | --- | --- |
| **Administrative information** | | |  |
| Title | 1 | Descriptive title identifying the study design, population, interventions, and, if applicable, trial acronym | 1 |
| Trial registration | 2a | Trial identifier and registry name. If not yet registered, name of intended registry | 3 |
|  | 2b | All items from the World Health Organization Trial Registration Data Set | 8 |
| Protocol version | 3 | Date and version identifier | 29 |
| Funding | 4 | Sources and types of financial, material, and other support | 32 |
| Roles and responsibilities | 5a | Names, affiliations, and roles of protocol contributors | 1, 32, 33 |
|  | 5b | Name and contact information for the trial sponsor | 32 |
|  | 5c | Role of study sponsor and funders, if any, in study design; collection, management, analysis, and interpretation of data; writing of the report; and the decision to submit the report for publication,  including whether they will have ultimate authority over any of these activities | 32 |
|  | 5d | Composition, roles, and responsibilities of the coordinating centre, steering committee, endpoint  adjudication committee, data management team, and other individuals or groups overseeing the trial, if applicable (see Item 21a for data monitoring committee) | 32 |
| **Introduction** |  |  |  |
| Background and rationale | 6a | Description of research question and justification for undertaking the trial, including summary of relevant studies (published and unpublished) examining benefits and harms for each intervention | 3-5 |
|  | 6b | Explanation for choice of comparators | 4, 5 |
| Objectives | 7 | Specific objectives or hypotheses | 6, 7 |
| Trial design | 8 | Description of trial design including type of trial (e.g., parallel group, crossover, factorial, single group), allocation ratio, and framework (e.g., superiority, equivalence, noninferiority, exploratory) | 7, 8 |
|  | 8.1 | State a) that the primary outcome is a surrogate endpoint, and b) the target outcome(s) whose intervention effect is being substituted for (can also be reported under item 6a, 7, or 8) | 6 |
| **Methods: Participants, interventions, and outcomes** | | |  |
| Study setting | 9 | Description of study settings (e.g., community clinic, academic hospital) and list of countries where data will be collected. Reference to where list of study sites can be obtained | 8 |
| Eligibility  criteria | 10 | Inclusion and exclusion criteria for participants. If applicable, eligibility criteria for study centres and  individuals who will perform the interventions (e.g., surgeons, psychotherapists) | 10-13 |

| Interventions | 11a | Interventions for each group with sufficient detail to allow replication, including how and when they will be administered | 13-15 |
| --- | --- | --- | --- |
|  | 11b | Criteria for discontinuing or modifying allocated interventions for a given trial participant (e.g., drug dose change in response to harms, participant request, or improving/worsening disease) | 14, 15 |
|  | 11c | Strategies to improve adherence to intervention protocols, and any procedures for monitoring adherence (e.g., drug tablet return, laboratory tests) | 20, 21, 25, 26 |
|  | 11d | Relevant concomitant care and interventions that are permitted or prohibited during the trial | 14, 15 |
| Outcomes | 12 | Primary, secondary, and other outcomes, including the specific measurement variable (e.g., systolic blood pressure), analysis metric (e.g., change from baseline, final value, time to event), method of  aggregation (e.g., median, proportion), and time point for each outcome. Explanation of the clinical relevance of chosen efficacy and harm outcomes is strongly recommended | 16-24 |
|  | 12.1 | State the practical or scientific reason(s) for using a surrogate endpoint as a primary outcome. | 17 |
|  | 12.2 | State what other surrogate endpoints were considered and why the current one(s) were chosen. | 17 |
|  | 12.3 | Justification for selected surrogate endpoint: a) evidence (or lack thereof) of surrogate endpoint validation; and b) evidence (or lack thereof) of validity being specific to the context used, e.g.,  intervention; disease; population. | 17 |
| Participant timeline | 13 | Time schedule of enrolment, interventions (including any run-ins and washouts), assessments, and visits for participants. A schematic diagram is highly recommended (see Figure) | Figure 2 |
| Sample size | 14 | Estimated number of participants needed to achieve study objectives and how it was determined,  including clinical and statistical assumptions supporting any sample size calculations | 9, 10 |
|  | 14.1 | Clarify if the sample size will be estimated to demonstrate that a minimum effect on the surrogate endpoint would be predictive of a benefit on the target outcome(s). | 9 |
| Recruitment | 15 | Strategies for achieving adequate participant enrolment to reach target sample size | 10-12 |
| **Methods: Assignment of interventions (for controlled trials)** | | |  |
| Allocation: |  |  |  |
| Sequence generation | 16a | Method of generating the allocation sequence (e.g., computer-generated random numbers), and list of any factors for stratification. To reduce predictability of a random sequence, details of any planned restriction (e.g., blocking) should be provided in a separate document that is unavailable to those who  enrol participants or assign interventions | 11 |
| Allocation  concealment mechanism | 16b | Mechanism of implementing the allocation sequence (e.g., central telephone; sequentially numbered, opaque, sealed envelopes), describing any steps to conceal the sequence until interventions are  assigned | 11 |
| Implementation | 16c | Who will generate the allocation sequence, who will enrol participants, and who will assign participants to interventions | 11 |

| Blinding (masking) | 17a | Who will be blinded after assignment to interventions (e.g., trial participants, care providers, outcome assessors, data analysts), and how | 11 |
| --- | --- | --- | --- |
|  | 17b | If blinded, circumstances under which unblinding is permissible, and procedure for revealing a participant’s allocated intervention during the trial | 11 |
| **Methods: Data collection, management, and analysis** | | |  |
| Data collection methods | 18a | Plans for assessment and collection of outcome, baseline, and other trial data, including any related processes to promote data quality (e.g., duplicate measurements, training of assessors) and a  description of study instruments (e.g., questionnaires, laboratory tests) along with their reliability and validity, if known. Reference to where data collection forms can be found, if not in the protocol | 16-25 |
|  | 18b | Plans to promote participant retention and complete follow-up, including list of any outcome data to be collected for participants who discontinue or deviate from intervention protocols | 24, 25 |
| Data  management | 19 | Plans for data entry, coding, security, and storage, including any related processes to promote data quality (e.g., double data entry; range checks for data values). Reference to where details of data  management procedures can be found, if not in the protocol | 25 |
| Statistical methods | 20a | Statistical methods for analysing primary and secondary outcomes. Reference to where other details of the statistical analysis plan can be found, if not in the protocol | 22-24 |
|  | 20b | Methods for any additional analyses (e.g., subgroup and adjusted analyses) | 23 |
|  | 20b.1 | State what the plans are to conduct subsequent analyses/studies to verify current findings on the target outcome(s). | 23 |
|  | 20c | Definition of analysis population relating to protocol non-adherence (e.g., as randomised analysis), and  any statistical methods to handle missing data (e.g., multiple imputation) | 23 |
| **Methods: Monitoring** | | |  |
| Data monitoring | 21a | Composition of data monitoring committee (DMC); summary of its role and reporting structure; statement of whether it is independent from the sponsor and competing interests; and reference to  where further details about its charter can be found, if not in the protocol. Alternatively, an explanation  of why a DMC is not needed | 32 |
|  | 21b | Description of any interim analyses and stopping guidelines, including who will have access to these interim results and make the final decision to terminate the trial | 32 |
| Harms | 22 | Plans for collecting, assessing, reporting, and managing solicited and spontaneously reported adverse  events and other unintended effects of trial interventions or trial conduct | 21, 22 |
|  | 22.1 | Comment on whether the trial design (including sample size and follow-up period), given the use of a surrogate endpoint, adequately captures the potential harms of the intervention being tested. | 21, 22 |
| Auditing | 23 | Frequency and procedures for auditing trial conduct, if any, and whether the process will be independent from investigators and the sponsor | 32 |

| **Ethics and dissemination** | | |  |
| --- | --- | --- | --- |
| Research ethics approval | 24 | Plans for seeking research ethics committee/institutional review board (REC/IRB) approval | 31 |
| Protocol  amendments | 25 | Plans for communicating important protocol modifications (e.g., changes to eligibility criteria,  outcomes, analyses) to relevant parties (e.g., investigators, REC/IRBs, trial participants, trial registries, journals, regulators) | 39 |
| Consent or assent | 26a | Who will obtain informed consent or assent from potential trial participants or authorised surrogates, and how (see Item 32) | 12, 13 |
|  | 26a.1 | State whether and how trial participants will be engaged and informed before enrolment that the trial  was designed to evaluate an intervention’s effect using a surrogate endpoint. | 12 |
|  | 26b | Additional consent provisions for collection and use of participant data and biological specimens in ancillary studies, if applicable | NA ‘No biologicial speciments were collected as part of this trial |
| Confidentiality | 27 | How personal information about potential and enrolled participants will be collected, shared, and  maintained in order to protect confidentiality before, during, and after the trial | 26, 31 |
| Declaration of interests | 28 | Financial and other competing interests for principal investigators for the overall trial and each study site | 32 |
| Access to data | 29 | Statement of who will have access to the final trial dataset, and disclosure of contractual agreements  that limit such access for investigators | 11 |
| Ancillary and post-trial care | 30 | Provisions, if any, for ancillary and post-trial care, and for compensation to those who suffer harm from trial participation | NA ‘there is no provision of ancillary care in this trial’ |
| Dissemination policy | 31a | Plans for investigators and sponsor to communicate trial results to participants, healthcare  professionals, the public, and other relevant groups (e.g., via publication, reporting in results databases, or other data sharing arrangements), including any publication restrictions | 26 |
|  | 31b | Authorship eligibility guidelines and any intended use of professional writers | 32 |
|  | 31c | Plans, if any, for granting public access to the full protocol, participant-level dataset, and statistical code | 31 |
|  | 31c.1 | If surrogate and target outcome data will be collected in the trial, state the open access arrangements for the data for future secondary research. | 31 |
| **Appendices** |  |  |  |
| Informed consent  materials | 32 | Model consent form and other related documentation given to participants and authorised surrogates | Additional files |

| Biological specimens | 33 | Plans for collection, laboratory evaluation, and storage of biological specimens for genetic or molecular analysis in the current trial and for future use in ancillary studies, if applicable | NA ‘No biologicial speciments were collected as part of this tria |
| --- | --- | --- | --- |

Please cite as: Manyara AM, Davies P, Stewart D, et al. Reporting of surrogate endpoints in randomised controlled trial protocols (SPIRIT-Surrogate): extension checklist with explanation and elaboration. *BMJ* 2024;386:e078525. doi:10.1136/bmj-2023-078525.
